# A Case of Unilateral Sweating of the Head

**Published:** 1875-06-15

**Authors:** Alonzo Bryan


### A CASE OF UNILATERAL SWEATING OF THE HEAD.

By Alonzo Bryan, M.D.

*From the Clinic, June 5, 1875.*

As every phenomenon that may perchance be of value in the investigation of the nervous system, is at present of great importance, I am glad to present for publication the account of a case of unilateral sweating of the head, having had the case for some time under observation. Only a few such cases have been observed, and it appears not with the care which their rareness would seem to demand. Dr. Bartholow, I believe, has reported several instances of the affection to the New York Neurological Society.

June 27, 1874.—Mrs. M. J. C. has unilateral sweating of the head. To-day I saw her when the temperature of the room was 92° Fah. The parts of the head and face to the right of the median line were sweating freely. The parts to the left were ordinarily moist, as is the skin during a state of common insensible perspiration. The temperature of the right and left temples differed to the extent of 1¼° Fah. under the following circumstances: A good medical thermometer was pressed upon the left temple a little back of the external angle of the eye (by applying over the bulb of the instrument, for the purpose of excluding the atmosphere, very thick folds of woollen flannel) and the temperature was found to be 98½° F. At the corresponding spot on the opposite or sweating side, the temperature was 97¼°. When the bulb was not covered in by flannel, in the still atmosphere, the relative difference of temperature was the same. The temperature at the bend of each elbow, with the arm flexed so as to cover in the bulb of the thermometer, was on each side alike, 99½° (the person had been taking active exercise). Within the mouth, on both sides, the temperature was uniform, exactly 100°. The touch, sensibility, and motility, were uniform and perfect. The two radial pulses were alike; when the patient was sitting against the back of a common chair, 88 pulsations in the minute. The sudoriferous glands only, were more active on the one side. The sweating extended very slightly to the left of the median line of the mouth and chin, but the median line of the forehead and nose was an exact line of demarcation. The difference of sweating did not extend below the head and face.

Only two pathological states could be ascertained to exist: unilateral neuralgia of the face and head, and psoriasis; the latter occurring in spots on the head and extremities. Contrary to what I had expected, the neuralgia was on the right side; *i. e.*, the sweating side. This latter point, to my surprise, I recently ascertained. I presume that in this case the functions of the affected sympathetic filaments are rather exalted instead of being depressed. It should be observed though that the transudation of perspiration seemed to be held in abeyance on the side unaffected with neuralgia. On the neuralgic side the degree of perspiration seemed to be that which ought normally to have been excited by the temperature to which the person was subject at the time of the examination.

The psoriasis may have been due to nervous influence also.

It may be observed in this case that only one vital action seemed to be interfered with: the transudation of perspiration. Touch, sensibility, and motility, were uniform and perfect upon the two sides. The special senses were perfect. The chemical and organic changes in the tissues did not seem to be retarded or hastened, as the temperature within the mouth was the same on either side, 100°. The difference of temperature of the two sides, externally, was doubtless owing to the difference of the cutaneous evaporation.

